# Acute kidney injury requiring dialysis after endovascular intervention for acute deep venous thrombosis: A case report and literature review

**DOI:** 10.1002/ccr3.9263

**Published:** 2024-09-25

**Authors:** Najlaa Essa A. H. Al‐Mannai, Dalal Sibira, Hissa Alsuwaidi, Ayman Elmagdoub, Elmukhtar Habas, Gamal Alfitori

**Affiliations:** ^1^ Department of Medical Education, Internal medicine Residency Program Hamad Medical Corporation Doha Qatar; ^2^ Department of Interventional Radiology Hamad Medical Corporation Doha Qatar; ^3^ College of Medicine, QU health Qatar University Doha Qatar; ^4^ Department of Internal medicine Hamad medical corporation Doha Qatar

**Keywords:** acute kidney injury, angioJet, deep vein thrombosis, pharmacochemical thrombectomy

## Abstract

**Key Clinical Message:**

Percutaneous precutaneous mechanical thrombectomy has been used for clot dissolution and removal in selected cases of iliofemoral deep vein thrombosis. Intravascular Hemolysis and hemoglobinuria caused by pharmachomechanical chather directed thrombolysis (PCDT) devices like the Angiojet is associated with an increased risk of acute kidney injury (AKI). Acute tubular necrosis that is severe enough to require hemodialysis can occur. Clinicians should be aware of this potential risk to ensure early recognition and timely referral to the nephrologist, and a clear explanation of the risk of AKI should be given to the patients undergoing this procedure.

**Abstract:**

Lower extremity deep vein thrombosis (DVT) is a frequently encountered medical condition, and one that can lead to death or major disability if not promptly treated. Anticoagulation alone may not always be enough for complete treatment. It has been reported that early thrombus removal can rapidly relieve symptoms and prevent disease progression in some selected cases. Percutaneous pharmacomechanical thrombectomy has been used for clot dissolution and removal in such cases. AngioJet is an increasingly used method of percutaneous mechanical thrombectomy for DVT that can cause intravascular hemolysis and potentially acute kidney injury (AKI). We report here a case of a 39 years old lady who developed severe AKI (illustrated by creatinine level of 664 μmol/L (7.5 mg/dL), bicarb of 13 mmol/L and being anuric), requiring hemodialysis secondary to intravascular hemolysis and hemoglobinuria that occurred immediately after the use of AngioJet pharmacomechanical catheter‐directed technique to treat an extensive iliofemoral DVT.

## 1 INTRODUCTION

Acute Deep venous thrombosis (DVT) is common and can lead to significant adverse outcomes. Hence, appropriate management should be initiated early. The main treatment of DVT is anticoagulation. However, due to extensive thrombosis, anticoagulation alone may not always be sufficient for treatment and complication prevention. More invasive interventions are available including catheter‐directed therapy (CDT), and percutaneous mechanical thrombectomy (PMT). Currently, CDT is being replaced by PMT devices like AngioJet rheolytic thrombectomy devices given better outcomes such as reducing the length of hospital stay, admission to intensive care and risk of post‐thrombotic syndrome (PTS).^1^, ^2^, ^3^, ^4^, ^5^ However, hemolysis has been noticed which is due to the way that AngioJet works. In which as a result will lead to hemoglobinuria and acute kidney injury (AKI). Few case reports about AKI following AngioJet rheolytic thrombectomy have been reported in the literature. We report a case of 39 years old lady who underwent AngioJet thrombectomy for acute iliofemoral DVT and developed AKI post‐procedure requiring hemodialysis (HD) secondary to intravascular hemolysis.

## 2 CASE PRESENTATION

A 39‐year‐old African lady with no known past medical history, was admitted with a 3‐day history of left leg pain and swelling.

**FIGURE 1:**
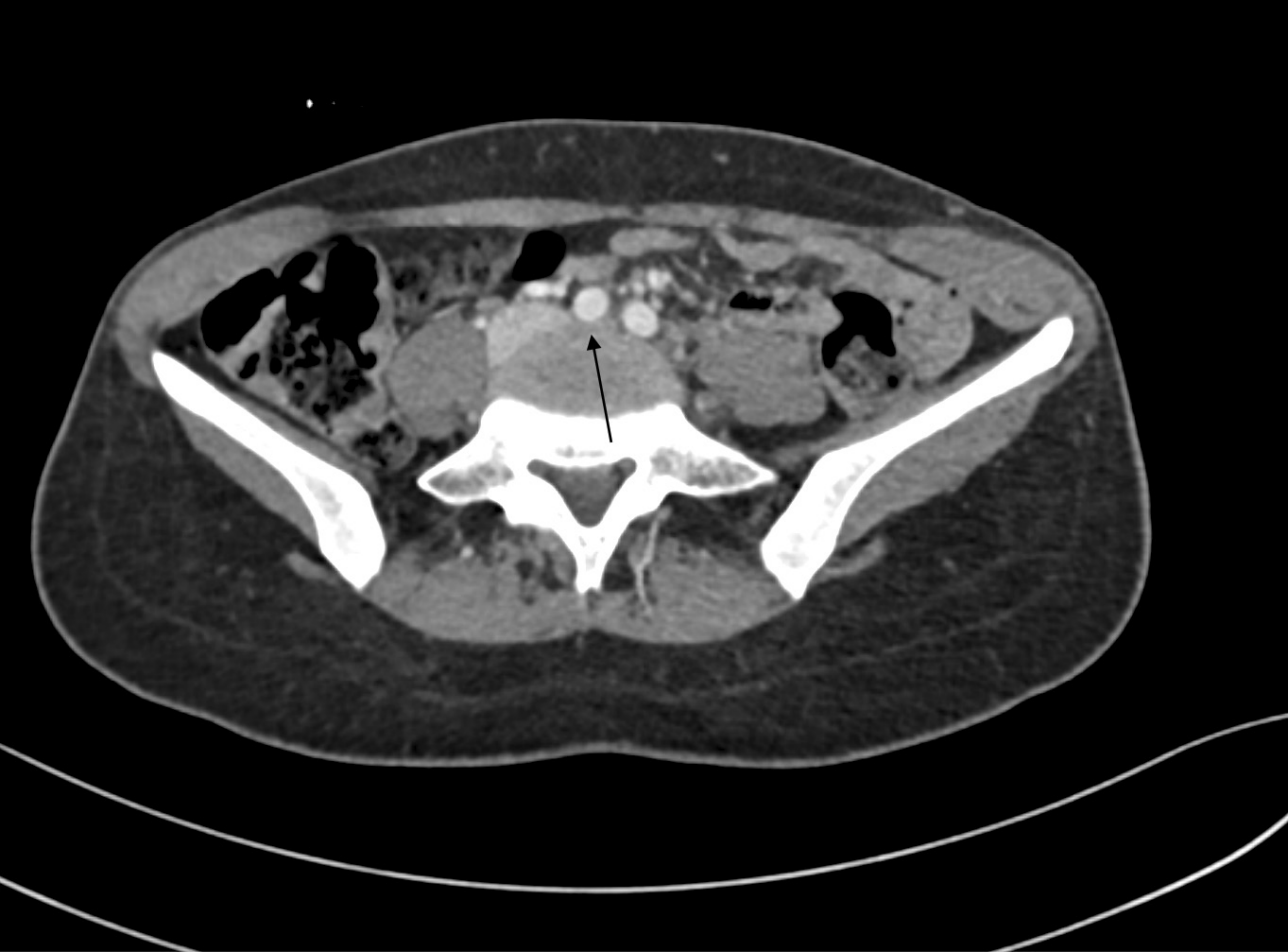
CT abdomen and pelvis with IV contrast (Venous Phase) at aortic bifurcation level, demonstrating the compression of the thrombosed Left common iliac vein (CIV) by the right CIA representing May Thurner Syndrome.

The patient reported having a fall around 14 days before her symptoms got started, and due to that her mobility was reduced. There was no history of chest pain, shortness of breath or palpitations. Examination revealed marked swelling of the left lower limb extending up the thigh with mild tenderness, but otherwise soft, no skin changes, and peripheral pulses were intact. Ultrasound doppler of the left lower limb was performed, which showed an extensive left lower limb DVT involving the common femoral vein, superficial femoral vein (SFV), popliteal vein, and proximal to mid posterior tibial veins. At this point, the interventional radiology (IR) team was involved and computed tomography abdomen and pelvis venogram was done upon their request. CT showed extensive venous thrombosis extending from the left SFV to the left common iliac vein (CIV) with radiological evidence of May Thurner syndrome (compression of the left CIV vein by the right common iliac artery) (Figure 1).

The patient was started on therapeutic low molecular weight heparin (LMWH); enoxaparin 1 mg/kg every 12 h and planned for IR intervention. Initial conventional venography runs showed no flow within the left deep venous system, accordingly, AngioJet machine used for thrombus clearance started by thrombectomy mode, followed by a pulse spray mode using 20 mg of Alteplase diluted in 180 mL of saline. The Alteplase was allowed to work for 30 min, then a further AngioJet thrombectomy was done followed by Venoplasty and balloon maceration (Total AngioJet time is 420 s).

**FIGURE 2:**
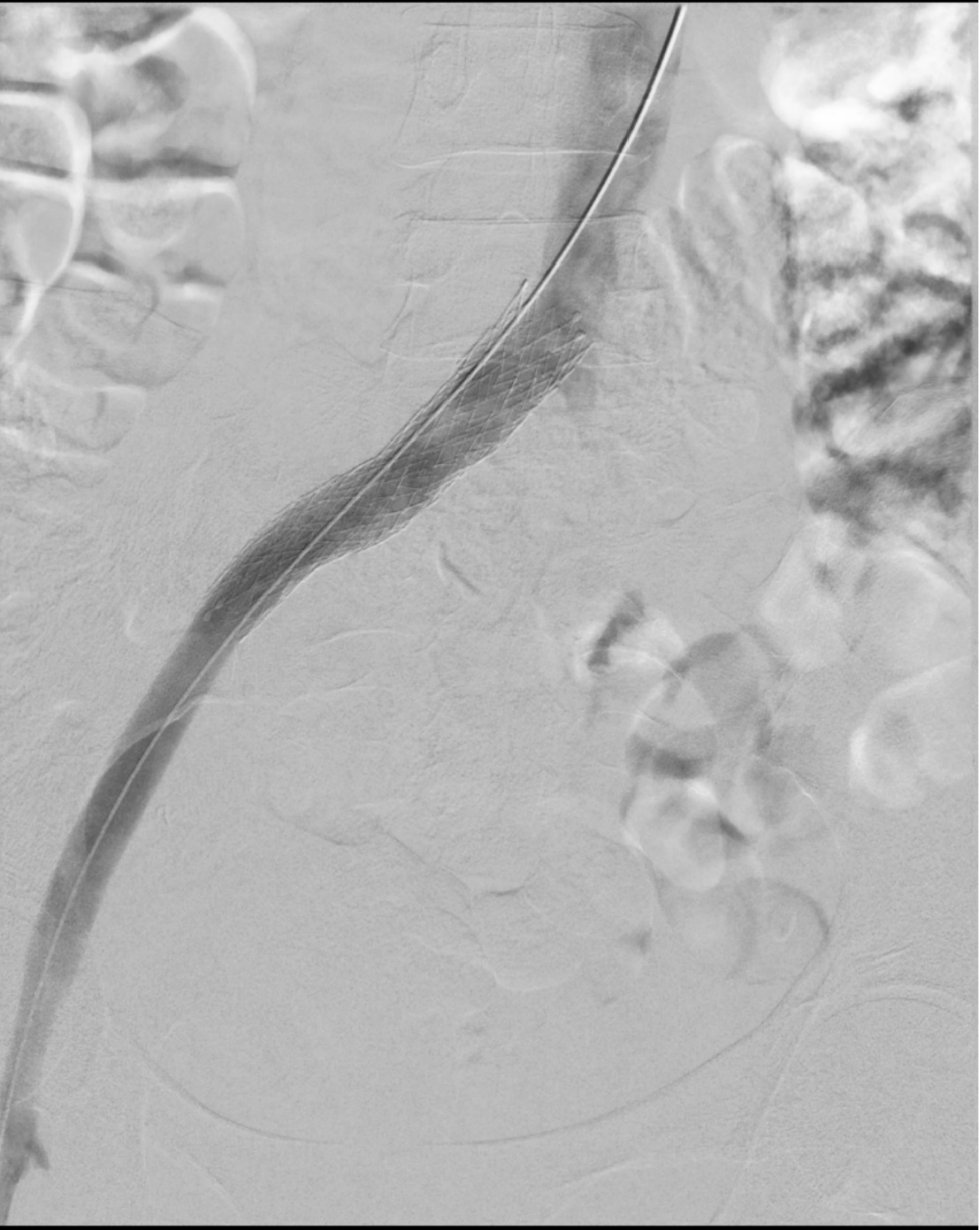
Showing a post stent placement venography, with optimal flow through the left external iliac vein, left common iliac vein (CIV) and IVC.

**FIGURE 3:**
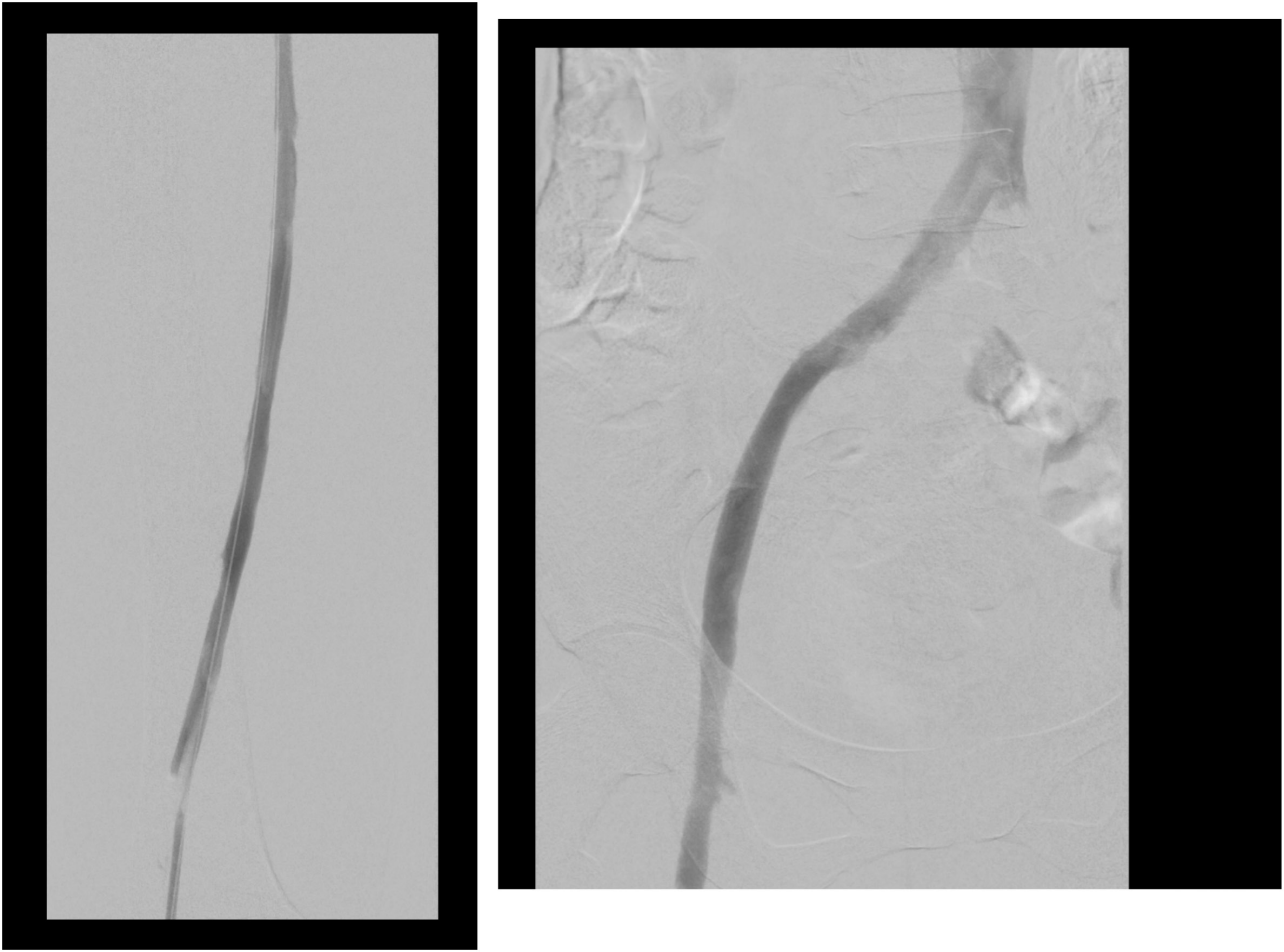
Post AngioJet thrombectomy/thrombolysis showing clearance of the left deep venous system of thrombosis.

Final contrast runs confirmed presence of May Thurner syndrome and appropriate stent placed in the stenotic segment of the left CIV (Figure 2). Post procedure venogram demonstrated optimal flow from the left SFV to the Inferior vena cava (75 mL of Omnipaque 300 mg/mL is used in this procedure) (Figure 3).

In the post‐procedural period, she developed vomiting and was treated with antiemetic and intravenous fluids. Following the surgical procedure, she was kept on therapeutic LMWH. Immediately after the intervention, her kidney function started to decline from baseline creatinine of 50 μmol/L (0.56 mg/dL) to 160 μmol/L (1.80 mg/dL).

## 3 METHODS

**FIGURE 4:**
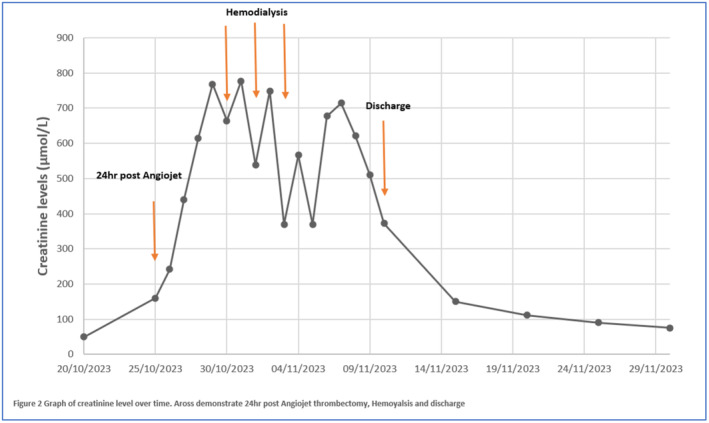
Graph of creatinine level over time.

Our differential diagnosis at this stage including pre renal AKI injury and contrast induced nephropathy. Then, she reported having red urine which on the urine dipstick tested positive for blood. Her serum amylase, lipase, Creatine kinase, and myoglobin were unremarkable. Kidney function continued to deteriorate over 72 h post‐operative (Figure 4). Laboratory investigation revealed a drop in hemoglobin and hematocrit levels from a baseline of 11.9 g/dL/36%–9.2 g/dL/23%. The lactate dehydrogenase (LDH) was high at 900 U/L. The direct antiglobulin test was negative. Blood tests performed pre‐intervention and within 72 h are listed in the Table 1.

**TABLE 1:** Laboratory investigations pre‐ and post‐Angiojet® thrombectomy.

Laboratory investigation	Pre‐procedure	Within 24 h post‐procedure	Within 96 h post‐procedure	Reference range
Hemoglobin	11.9	10.8	7.9	12.0–15.0 gm/dL
White blood cells	4.7	5.6	5.8	4–11 × 10^9^
Platelets	260	220	180	150–400 × 10^9^
Creatinine	60	160	750	44–80 μmol/L
Estimated glomerular filtration rate	88	33	7	>90 mL/min
Urea	3.6	7.1	27	2.5–7.8 mmol/L
Potassium	3.5	4.6	5.2	3.5–5.3 mmol/L
Sodium	140	136	137	133–146 mmol/L
Bicarbonate	27	21	13	22–29 mmol/L
Albumin	31	27	22	35–50 gm/L
Bilirubin	4	3	5	0–21 μmol/L
Myoglobin	_	80	_	25–58 ng/mL
CK	_	50	_	2–238 U/L
International normalized ratio	1.2	1.0	1.0	0.8–1.2 Ratio
Activated partial thromboplastin time	31	32	54 (on heparin infusion)	25–36.5 s
Lactate dehydrogenase	_	901	840	135–214 U/L
Direct antiglobulin test	_	_	negative	_
Peripheral smear	Red cells: moderate normochromic normocytic anemia, few spherocytes, and mild rouleaux formation.			_
Urine microscopy	Urine RBCs 33, WBC 9			
Urine dipstick	2+ protein, +3 blood			

All investigations for AKI including autoimmune, virology, and even US kidney and renal doppler were unremarkable. Kidney function continued to decline over 4 days post‐procedure, creatinine levels reached to 664 μmol/L (7.5 mg/dL) and she became anuric along with severe metabolic acidosis not responding to IV normal saline and sodium bicarbonate infusion. This necessitated dialysis Catheter insertion through the right internal jugular vein and initiation of intermittent renal replacement therapy. After three sessions of HD, her kidneys started to show signs of recovery with polyuria, passing around 3–4 L of clear urine per day. Her renal function continued to improve, and her creatinine level was trending down. Due to the decline of kidney function, anticoagulation was initially changed to heparin infusion and consequently, warfarin was started when the renal function improved. She was discharged after fully recovered and the therapeutic INR was achieved.

## 4 DISCUSSION

Venous thromboembolism remains a major health problem with its recognized risk of sudden death secondary to pulmonary embolism and the risk of significant morbidity due to the development of PTS. PTS is associated with reduced quality of life and a substantially increased economic burden.^3^, ^4^

Anticoagulation alone may not always be enough for complete treatment. Endovascular interventions, in this regard, have emerged as a key role player in preventing PTS or, when chronic, relieving symptoms. The outcomes from trials like CaVenT (catheter‐directed thrombolysis in acute iliofemoral vein thrombosis), ATTRACT (acute venous thrombosis: thrombus removal with adjunctive catheter‐directed thrombolysis), and the more recent CLOUT study (a large prospective registry for DVT) underscore the benefits of endovascular thrombus removal for acute iliofemoral deep vein thrombosis. These trials showed improvements in vein patency, reduced risk of post‐thrombotic complications, and significant improvement in symptoms and health status over months, thereby supporting the effectiveness and safety of mechanical thrombectomy in these cases.^1^, ^2^, ^20^

The Pharmacomechanical catheter‐directed thrombolysis (PCDT) devices have been used for clot dissolution and removal in selected cases. These devices like the Angiojet can simultaneously macerate the thrombus and infuse the thrombolytic agent. The AngioJet Rheolytic Thrombectomy System utilizes saline jets to macerate the thrombus and can reduce lytic dose and exposure compared to catheter‐directed thrombolysis (CDT) alone.^5^, ^6^, ^7^ Although effective, this technique, however, has been associated with the development of AKI owing to the intravascular hemolysis and the resultant hemoglobinuria caused by these devices, mainly the AngioJet.^8^ Although this risk of AKI has become an increasingly recognized complication of Angiojet, it remains underappreciated in clinical practice. Morrow et al. found the incidence of AKI in patients with arterial and venous thromboses, undergoing PCDT with Angio‐Jet to be significantly higher in the PCDT group compared to CDT controls, 21% and 0% (*p* = 0.033), respectively. None of the PCDT patients however required renal replacement therapy.^11^ Similarly, Escobar et al. observed AngioJet to be an independent risk factor for the development of AKI in 29% of patients while only (8%) of the CDT group had AKI (odds ratio 8.22, *p* = 0.004). Only two patients (4%) required dialysis in the Angiojet group and none in the CDT group.^12^ The risk of postprocedural AKI in patients with acute iliofemoral deep venous thrombosis (IFDVT) who underwent percutaneous pharmacomechanical thrombectomy using AngioJet was investigated in a retrospective study carried by Yang Shen and his colleagues; who found out that a history of major surgery within 3 months of endovascular intervention was an independent risk factor that raise the risk of postprocedural AKI.^8^ In another study; Salem and his colleagues found through their retrospective comparative review that bilateral deep vein thrombosis (DVT) and being female were significant factors associated with an increased risk of AKI in patients who underwent pharmacomechanical thrombolysis with the AngioJet device.^16^ These authors however did not include thrombectomy volume or time as part of their analysis.

The occurrence of intravascular hemolysis in our patient, as evidenced by the urine and laboratory investigations, was an expected consequence of the procedure. But, given the patient's young age and the absence of other risk factors, the deterioration in her renal function to the point of requiring HD (Figure 3), was not anticipated. The patient had a venogram intra‐operatively which involves administering a small dose of contrast and she developed significant vomiting post‐operatively, both of which could have contributed to AKI. However, the speed and severity of the development of the AKI in her case, despite aggressive fluid management, suggests the cause of deterioration in renal function was likely intravascular hemolysis, as previously reported.^19^

Intravascular hemolysis is a recognized cause of AKI in many conditions, including autoimmune hemolysis, paroxysmal nocturnal hemoglobinuria, snake and insect bites (envenomation) and hemolysis secondary to prosthetic cardiac valves.^9^, ^10^ The AKI is caused by the nonprotein heme pigment that is released from the hemoglobin and is toxic to the kidney. The heme pigment may damage the kidney either by the tubular obstruction, possibly in association with uric acid, by direct proximal tubular epithelial cell injury, heme‐induced renal vasoconstriction or through pigment cast formation in the renal tubules.^13^, ^14^ Prevention and management of AKI associated with hemolysis is an area which requires further investigation. The use of sodium bicarbonate has shown potential benefits through the alkalinization of urine and the reduction of oxidative stress on renal tubules.^15^

Many previous cases suggested that the short‐term prognosis of patients who developed AKI post‐AngioJet is good. According to previous reports, it can take around 3–12 months for full renal recovery post‐AngioJet.^17^, ^18^ However, more studies need to investigate the long‐term prognosis of patients who had AKI requiring renal replacement therapy post AngioJet thrombectomy.

## 5 CONCLUSION

Hemolysis and hemoglobinuria caused by pharmacomechanical catheter‐directed thrombolysis (PCDT) devices is associated with an increased risk of AKI. Acute tubular necrosis that is severe enough to necessitate renal replacement therapy can happen. Consideration should be given to pre‐hydration for patients undergoing an AngioJet procedure, particularly when additional risk factors for AKI are present. Raising awareness among clinicians about the potential risk of AKI post‐AngoJet is crucial. This heightened vigilance enables early recognition and facilitates timely referral to nephrology services. A clear explanation of the potential risk of AKI should be given to the patients undergoing this procedure.

## AUTHOR CONTRIBUTIONS

**Najlaa Essa A. H. Al‐Mannai:** Writing – original draft; writing – review and editing. **Dalal Sibira:** Writing – original draft. **Hissa Alsuwaidi:** Writing – review and editing. **Ayman Elmagdoub:** Supervision; writing – review and editing. **Elmukhtar Habas:** Supervision; writing – review and editing. **Gamal Alfitori:** Supervision; writing – review and editing.

## FUNDING INFORMATION

This article is funded by the Qatar National Library whom they have no role in manuscript writing.

## CONFLICT OF INTEREST STATEMENT

All authors declare no conflicts interest.

## CONSENT

Written informed consent was obtained from the patient to publish this report in accordance with the journal's patient consent policy.

## Data Availability

The data that support the findings of this study are available from the corresponding author upon reasonable request.
